# Comparative Evaluation of Bone Repair with Four Different Bone Substitutes in Critical Size Defects

**DOI:** 10.1155/2020/5182845

**Published:** 2020-05-23

**Authors:** Gustavo Grossi-Oliveira, Leonardo P. Faverani, Bruno Coelho Mendes, Tárik Ocon Braga Polo, Gabriel Cury Batista Mendes, Valthierre Nunes de Lima, Paulo Domingos Ribeiro Júnior, Roberta Okamoto, Osvaldo Magro-Filho

**Affiliations:** ^1^Oral and Maxillofacial Surgery, Diagnostic and Surgery, Aracatuba School of Dentistry, Sao Paulo State University (UNESP), Aracatuba, Sao Paulo, Brazil; ^2^Oral and Maxillofacial Surgery, Department of Oral Surgery, University of Sagrado Coracao, Bauru, Sao Paulo, Brazil; ^3^Human Anatomy, Department of Basic Sciences, Aracatuba School of Dentistry, Sao Paulo State University (UNESP), Aracatuba, Sao Paulo, Brazil

## Abstract

This study evaluated the osteoconductive potential of four biomaterials used to fill bone defects. For this, 24 male Albino rabbits were submitted to the creation of a bilateral 8 mm calvarial bone defect. The animals were divided into four groups—bovine hydroxyapatite, Bio-Oss® (BIO); Lumina-Bone Porous® (LBP); Bonefill® (BFL); and an alloplastic material, Clonos® (CLN)—and were euthanized at 14 and 40 days. The samples were subjected to histological and histometric analysis for newly formed bone area. Immunohistochemical analysis for Runt-related transcription factor 2 (Runx2), vascular endothelial growth factor (VEGF), and osteocalcin (OC) was performed. After statistical analysis, the CLN group showed greater new bone formation (NB) in both periods analyzed (*p* < 0.05). At 14 days, the NB showed greater values in BIO in relation to LBP and BFL groups; however, after 40 days, the LBP group surpassed the results of BIO (*p* < 0.001). The immunostaining showed a decrease in Runx2 intensity in BIO after 40 days, while it increased for LBP (*p* < 0.05). The CLN showed increased OC compared to the other groups in both periods analyzed (*p* < 0.05). Therefore, CLN showed the best osteoconductive behavior in critical defects in rabbit calvaria, and BFL showed the lowest osteoconductive property.

## 1. Introduction

The use of dental implants for oral rehabilitation of partial or total edentulous patients has become a promising therapeutic modality in recent decades, returning to patients the functions of the stomatognathic system by masticatory restoration and aesthetics [1, 2].

After teeth loss, a physiological process takes place which leads to bone resorption of the alveolar ridge in thickness and height, becoming a limiting factor for the placement of dental implants, without prior reconstruction of the region with bone grafts [1, 2].

Autogenous bone graft is still considered to be the “gold standard” because it has osteogenic, osteoinductive, and osteoconductive properties [1–3]. To overcome the disadvantages of autogenous bone removal in performing bone reconstruction, new materials are being developed in order to assist bone repair in regions where the intention is to maintain or restore bone volume [4]. These biomaterials are designed to maintain the three-dimensional framework in the region to be repaired and develop an osteoconductive surface structure leading to migration of osteoblasts and deposition of bone matrix [5]. Although bone substitutes have the same purpose of dimensional maintenance and osteoconductive surface, the quality of bone formation may differ in physicochemical characteristics depending on the bone substitute applied. It is possible to argue, then, that the manufacturing method is responsible for the properties conferred on the biomaterial, and therefore, its ability to interact with the native bone [6].

Bio-Oss ® (BIO) is a bone substitute obtained from bovine bone, which favors the proliferation of blood vessels and bone cells migration through the coarse, meshed, and interconnecting pore system in accordance with the manufacturer (Bio-Oss ®). There are a great number of studies indicating it as a bone substitute capable of achieving an excellent standard of bone formation in maxillofacial region [7, 8]. As Bio-Oss®, Lumina-Bone Porous® and Bonefill® are xenogenous biomaterials composed of bovine mineral bone, which act in a way to favor osteoconduction. Still, the Clonos® ceramic biomaterial, of alloplastic origin, is characterized by the biphasic composition between calcium phosphate and hydroxyapatite and is used to fill bone defects as a substitute. These biomaterials are used as an alternative to autogenous bone, offering support for cell migration and bone neoformation, according to the manufacturers.

Due the notorious absence of scientific evidence among the biomaterials, this study aims to elucidate the bone formation and osteoconductive potential in critical defects at calvaria of rabbits using three bone substitutes compared to the Bio-Oss ® (positive control) in periods of 14 and 40 days. The hypothesis is that there was difference in the parameter analyzed between experimental groups, with probable superiority of Bio-Oss®, and the null hypothesis is that there was no statistical difference between the groups.

## 2. Materials and Methods

### 2.1. Animals

This study was approved by the Ethics Commission on Use of Animals, process 18/14. Twenty-four Albino male rabbits (Genetic Group of Botucatu, São Paulo, Brazil), at 3 to 4 months of age and weighing between 3.5 and 4 kg, were used for this research. These animals were distributed in individual cages with standard diet—solid feed (Pro Rabbit, Primor, São Paulo, SP, Brazil) and water *ad libitum*, during the experimental time.

### 2.2. Experimental Design and Surgical Procedure

The animals were randomly divided into four groups, *n* = 6, specified according to the bone substitute used to fill in the defect: Bio-Oss (BIO), Lumina-Bone Porous (LBP), Bonefill (BFL), or Clonos Dental. The experimental periods used were 14 and 40 days. Eight hours prior to surgery, the animals were fasted. They were sedated by the combination of 50 mg/kg intramuscular ketamine (IM) (Vetaset—Fort Dodge Animal Health Ltd., Campinas, São Paulo, Brazil) and 5 mg/kg xylazine hydrochloride (Dopaser—Calier Laboratory of Brazil Ltd., Osasco, São Paulo, Brazil). The trichotomy in the calvaria region was held, and subsequently antisepsis was performed with iodine polyvinylpyrrolidone (Rioquímica, São José do Rio Preto). Local infiltration with mepivacaine 2% and epinephrine 1 : 200,000 (DFL, Industry & Trade SA, Rio de Janeiro, Brazil) was also performed. All procedures were performed by the same surgeon.

A linear incision at the sagittal plane was made with 4 cm of extension. The incision initially involved the skin, subcutaneous tissue, muscle and aponeurotic occipitofrontal galea, followed by pericranium, and detachment with subsequent calvaria exposure. A trephine drill of 8 mm, at outside diameter, mounted in a reducing hand piece (20 : 1) and connected to an electric motor (BLM 600 plus, Driller, Jaguaré, SP, Brazil) with controlled rotation at 1200 rotation per minute, was used under 0.9% saline irrigation [9, 10] to perform the critical defect and remove the total thickness bone blocks [5, 11, 12]. Soon after, the defects were filled with heterogeneous biomaterials according to the group, moistened in saline solution 0.9%. Internal sutures were performed with Polyglactin 910 4-0 (Vicryl 4.0, Ethicon, Johnson Prod., São José dos Campos, Brazil) and external sutures with Nylon 4.0 (Ethicon, Johnson, Sao José dos Campos, Brazil). Postoperatively, the animals received a single dose of intramuscular antibiotic (0.1 ml/kg, Fort Dodge Animal Health Ltd., Campinas, São Paulo, Brazil) and tramadol (1 mg/kg/day, Ariston Chemical and Pharmaceutical Industries Ltd., São Paulo, Brazil) over 3 days. Twelve animals were euthanized with a lethal dose of pentobarbital sodium (200 mg/kg) at 14 and 40 days after surgery. The calvariae were extracted and reduced with an oscillating saw, maintaining 2 mm anterior and posterior margin to the bone graft.

### 2.3. Laboratory Processing

The pieces were fixed in 10% buffered formalin (Reagents Analytical, Dental-Hospital Dynamics Ltd., Catanduva, SP, Brazil) for 48 hours, rinsed in water for 24 hours, decalcified in ethylenediaminetetraacetic acid (10%), and then dehydrated using an alcohols sequence. After these steps, the clarification with xylene was carried out for later inclusion in paraffin. Semiserial cuts of 5 *μ*m thickness were prepared and stained with hematoxylin and eosin (HE) for histometric analysis, Masson Mallory [13], and others for the immunohistochemical reactions.

Prior to performing the histological and immunohistochemical analysis, the samples were coded, so that only the supervisor knew to which groups they belonged.

### 2.4. Histological Analysis

The images were captured on a conventional optical microscope (Leica Microsystems Aristoplan Leitz, Bensheim, Germany) linked to a capture camera (Leica DFC 300FX, Leica Microsystems, Heerbrugg, Switzerland) and connected to a microcomputer with Axio Vision 4.8 software (Carl Zeiss, Oberkochen, Germany).

Histological evaluation consisted of the investigation of the events that occurred at 14 and 40 days and the relationship of the new bone formation with the particles of substitutes used to fill the 8 mm bone defects, similar to the trephine diameter.

### 2.5. Histometric Analysis

After staining the slides with hematoxylin (Merck & Co., Inc., Germany), measurements were performed using the same optical microscope (Leica DMLB, Heerbrugg, Switzerland) coupled to an image capture camera (Leica DC 300F Microsystems Ltd., Heerbrugg, Switzerland) and connected to a microcomputer. A calibrated author, using the remaining bone segments as a reference, located the central region of the defects. Thus, three images of each defect in 40x objective were acquired and saved in TIFF files. Finally, the images were assessed using the image analyzer software ImageJ (Image Processing and Analysis Software, Ontario, Canada).

The histometric analysis was performed by calculating the present bone area in the center of bone defects. Therefore, prior to analysis, the program was calibrated using a calibration ruler that was photographed under a microscope with the same objective lens of histological slides (original X40 magnification). Thus, the newly formed bone area was measured (NB) in *μ*m [2], in the three captured images, and added to obtain the mean, for the different biomaterials used and experimental periods (14 and 40 days).

### 2.6. Immunohistochemical Analysis

The immunohistochemical reaction was performed through detection by immunoperoxidase. After decalcification of histological sections, the activity of endogenous peroxidase was inhibited by hydrogen peroxide. Next, the slides passed through the antigen retrieval step with citrate phosphate buffer (pH 6.0), blocking the endogenous biotin using milk. Primary antibodies against Runx2 (sc-101145), VEGF (sc-7269), and OC (sc-365797) were used (Santa Cruz Biotechnology, Dallas, Texas, USA) [13].

The biotinylated anti-goat secondary antibody produced in donkeys (Jackson Immunoresearch Laboratories) was used, and the amplifiers selected were avidin and biotin (Vector Laboratories, Burlingame, CA). Diaminobenzidine (Dako, Glostrup Denmark) was used as chromogen. The counterstaining of histological sections was performed by Harris hematoxylin. For each of the antibodies used, the expression of these was assessed semiquantitatively by assigning different proteins “scores” according to the immunomarked area in the bone repair process. The analysis was conducted under an optical microscope (Leica DMLB, Heerbrugg, Switzerland), and the intensity area of immunostaining was assessed semiquantitatively with scores from 0 to 3: 0, absence of immunostaining; 1, light immunostaining (around 25% of labeling area); 2, moderate immunostaining (around 50% of labeling area); and 3, intense immunostaining (around 75–100% of labeling area) [13, 14].

### 2.7. Statistical Analysis

Comparing the histometric data (NB and immunohistochemistry scores), the Shapiro–Wilk normality test was applied, and homogeneity of the results was observed (*p* > 0.05). Comparisons of individual interactions (bone substitutes, time) and bone substitutes interaction versus time were performed by 2-factor variance analysis, ANOVA. Values showed statistical significance applying the Tukey test. All tests were performed in the SigmaPlot 13.0 statistical program (Scientific Graphing and Data Analysis Software, San José, CA, USA), which used as a significance level *p* < 0.05.

## 3. Results

### 3.1. Qualitative Analysis (14 and 40 days)

#### 3.1.1. 14 Days

**Figure 1:**
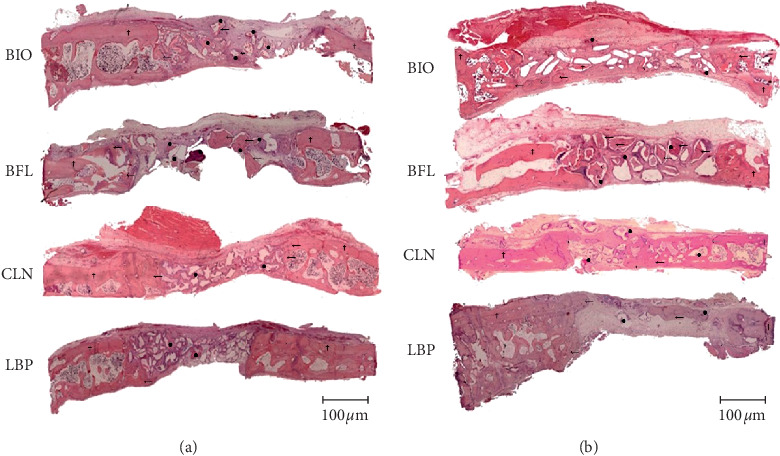
Histological panoramic images of bone defects filling biomaterials (BIO, BFL, CLN, and LBP), in the experimental periods of 14 and 40 days postoperatively (HE staining, original magnification 63x). Lamellar bone (†), newly formed bone (←), and remaining biomaterial (●). (a) 40 days. (b) 14 days.

**Figure 2:**
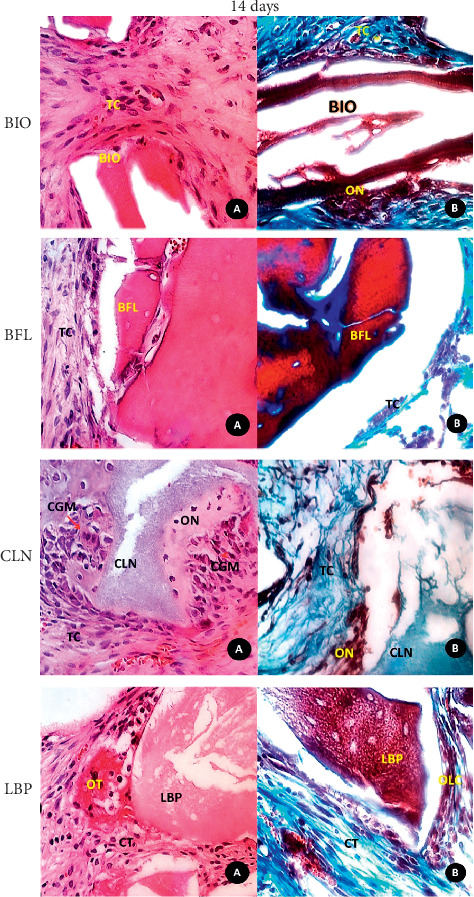
Photomicrograph of histological sections: (A) histological detailed features of the biomaterials (BIO, BFL, CLN, and LBP) and surrounding tissues in the central region of the defect at 14 days (HE, original magnification 40x); (B) mallory trichrome staining (original magnification 40x). CT: connective tissue; NB: newly formed bone; OLC: osteoblastic line cells; OT: osteoid tissue.

BIO: on the periphery of the defect, there were granules of biomaterial surrounded by newly formed bone tissue and active osteogenesis within richly cellularized and vascularized granulation tissue. In the central region of the defect, there were biomaterial granules through the loose connective tissue moderately cellularized and rich in blood vessels (Figures 1 and 2).

BFL: the biomaterial fragments showed a nonviable cortical bone tissue aspect, which was permeated by richly cellularized granulation tissue or loose connective tissue. Osteogenic activity or bone formation in contact with the biomaterial was not observed. Peripherally, newly formed bone was observed in contact with the defect walls (Figures 1 and 2).

CLN: irregular pieces of biomaterial through the richly cellularized granulation tissue were observed. On their surfaces, discreet and direct deposition of newly formed bones and intense osteoblastic activity were visible (Figures 1 and 2).

**Figure 3:**
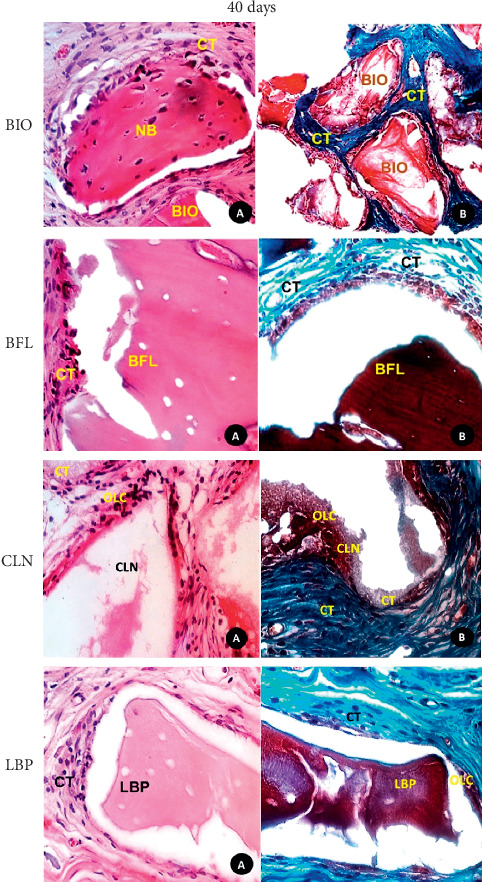
Photomicrograph of histological sections: (A) histological detailed features of the biomaterials (BIO, BFL, CLN, and LBP) and surrounding tissues in the central region of the defect at 40 days (HE, original magnification 40x); (B) mallory trichrome staining (original magnification 40x). CT: connective tissue; NB: newly bone formed; OLC: osteoblastic line cells. In CLN: (a) Mallory trichrome staining, (b) HE, original magnification 40x.

LBP: the biomaterial granules were present in various sizes, some slightly rounded. Between the particles, cellular connective tissue and the absence of bone formation were observed (Figures 1 and 3).

#### 3.1.2. 40 Days

BIO: after 40 days, there was trabecular bone in maturation phase with rounded and isolated edges. With the evolving of the biomaterial granules, there was predominance of fibrous connective tissue with mild diffuse mononuclear cell infiltration, as well as mature adipose tissue foci. Foreign body reaction was noted by the presence of multinuclear giant cells (MGCs) on the biomaterial surface (Figures 1 and 3).

BFL: there were biomaterial fragments permeated by fibrous connective tissue showing foci of intense mononuclear inflammatory infiltrate and surrounded by MGCs associated mononuclear leukocytes (Figures 1 and 3).

CLN: there was remodeling lamellar bone tissue surrounding the biomaterial particles, marked by numerous reversal lines. In the central region of the defect, there were also loose fibrous connective tissue and eventual osteogenesis regions. A strange reaction body type was noted by the presence of numerous MGCs on the beads (Figures 1 and 3).

LBP: Few biomaterial granules surrounded by loose fibrous connective tissue and adipose tissue foci were observed, without associated bone formation. Mature trabecular bone through the central defect in the remodeling phase was observed. Active regions of osteogenesis were also noted (Figures 1 and 3).

### 3.2. Histometric Analysis

Regardless of the interactions (assessed/isolated biomaterials, time, or biomaterials interaction vs time), all associations were statistically significant (*p* < 0.001, 2-factor ANOVA) (Table 1).

When analyzing the average percentage of bone neoformation independently of time, the BIO and LBP groups showed similar behavior. The other groups showed significant differences in comparison (*p* < 0.001) (Table 2).

**Figure 4:**
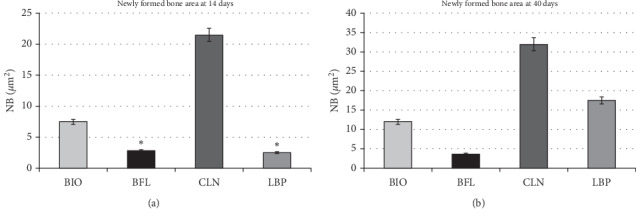
Representative charts showing average values and standard deviation of newly formed bone area of the experimental groups (BIO, BFL, CLN, and LBP) at 14 and 40 days postoperatively with *p* < 0.001, except for BFL and LBP at 14 days, which showed no difference (^*∗*^).

When assessing intragroup data, all bone substitutes showed statistically significant differences, comparing the periods of 14 and 40 days (*p* < 0.001) (Figure 4).

When observing the bone formation at 14 days, the CLN group showed higher values than the other groups (21.62 ± 1.23). The BIO group had a mean of 7.52 ± 0.27 percentage bone formation, the LBP group 2.55 ± 0.3, and the BFL group 2.85 ± 0.56. Comparing the biomaterials values, we found no statistical difference between LBP and BFL; other interactions showed significant differences (*p* < 0.001) (Table 3) (Figure 4).

At 40 days, the values were 32.29 ± 1.2 for CLN, 12.38 ± 0.73 for BIO, and 17.75 ± 0.78 to 3.92 ± 0.3 for LBP and BFL. All interactions between groups presented statistically significant differences (*p* < 0.001, Tukey test) (Table 4) (Figure 4).

### 3.3. Immunohistochemistry Analysis

#### 3.3.1. 14 Days

**Figure 5:**
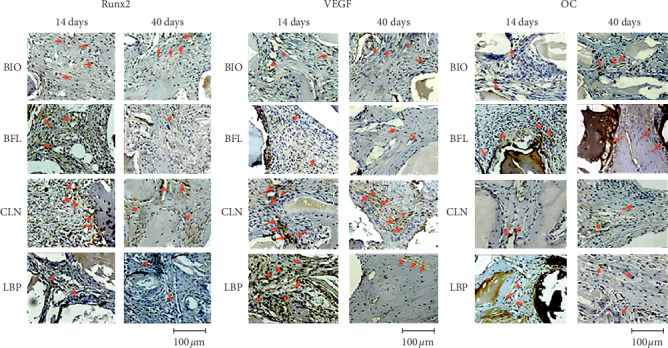
Representative images of the Runx2, VEGF, and OC immunostaining intensity (red arrow) in the experimental groups BIO, BFL, CLN, and LBP at 14 and 40 days postoperatively (original magnification 40x).

Representative values of scores in relation to the intensity of immunostaining of RUNX 2 protein, VEGF, and OC for each group can be viewed in Table 5 and are presented in Figure 5.

Immunostaining for RUNX 2 protein was lightly marked (1) for the LBP and BIO groups, while the BFL and CLN groups were moderately labeled (2). For VEGF, only the group BIO presented light immunostaining (1), while the other groups had moderate immunostaining (2). As for OC, the LBP, BFL, and BIO groups showed moderate labeling (2), and only the CLN group was lightly marked (1).

#### 3.3.2. 40 Days

For RUNX 2, LBP, BFL, and CLN groups were moderately marked (2), and only the BIO group was slightly marked (1). For VEGF, the CLN group had moderate immunostaining (2), while the other groups showed slight immunostaining (1). As for the OC protein, LBP had only slight marking (1), and the other groups had moderate marking (2).

After application of 2-factor ANOVA (group vs periods) and the Tukey posttest, the scores obtained by immunohistochemical analysis were observed for each protein, and the following interactions were determined to be statistically significant or not.

For RUNX 2, the immunostaining intragroup changes were significant only in the LBP group (*p* < 0.05, Tukey test). In the intergroup analysis, at 14 days, statistical significance was noted in the interactions of the CLN vs. BFL groups, BFL vs. BIO, BFL vs. LBP, and LBP vs. BIO. And at 40 days, only the comparison of LBP vs. BIO groups was significant (*p* < 0.05, Tukey test).

The VEGF protein showed a change in the comparison of 14 vs. 40 days only in the LBP and BFL groups (*p* < 0.05, Tukey test). At 14 days, the LBP vs. BIO interaction showed a significant change (*p* < 0.05, Tukey test), and at 40 days, no differences were found between the tested groups (*p* < 0.05, Tukey test).

For OC, it was noted that, in both intragroup and intergroup analysis, all interactions showed significant changes (*p* < 0.001, Tukey test).

## 4. Discussion

The hypothesis presented by the study was that, with regard to the bone formation in the critical defects, different results were expected in the experimental groups, with enhanced results in the BIO group, and this was partially accepted. There were statistical differences between groups in both experimental times (*p* < 0.005); however, independently of time, higher values were observed in NB with CLN biomaterial (*p* < 0.001).

In this animal model used to evaluate the osteoconductive potential of biomaterials, a bone defect considered critical was adopted, which means that it could not heal by itself without the filling of any bone substitute until the longest experimental period. In this context, the defect created, with 8 mm of diameter, and the periods of euthanasia, of 14 and 40 days, were favorable to analyze the osteoconductive potential of biomaterials [5, 11, 12]. Furthermore, studies on the behavior of bone substitutes indicate the calvaria of rabbit as a recommended anatomical region for this type of analysis due to the absence of forces or mechanical stress and similarity to the maxilla in terms of blood supply and medullary component [5, 11, 12, 15, 16]. Among the bone substitutes used in alveolar ridge reconstruction, Bio- Oss ® is a material composed of bovine hydroxyapatite and having the most clinical and scientific evidence in the literature [17–19]. Demonstrably, BIO is indicated in filling circumferential defects, fenestrations around dental implants, and sinus lift technique in the posterior maxilla [20, 21]. It is known that, in the clinical setting, the reconstructions of large magnitude bone defects, usually caused after resection of tumors, facial trauma with loss of bone substance, or congenital defects, are hardly successfully treated with isolated bone substitutes [20, 21]. This was observed in the results obtained in the present research, given that even Bio-Oss®, with very interesting results in bone reconstruction, showed lower values for the parameters analyzed in osteoconductive performance compared to other biomaterials (CLN and LBP).

Similarly, as found in the present study, Tovar et al. [22] also observed reduced NB in critical defects created on the calvaria of rabbits filled with BIO, against initial expectations. It was clear that until the last time period assessed, BIO kept filling the bone defect in all its extension. Initially, it did so with active osteogenic activity, and then with the formation of organized tissue. These findings are corroborated by histometry and also by immunostaining of the Runx2 transcription factor, which shows the preosteoblast activity and confirms that BIO, though it may be a good bone substitute in small and medium defects, needs a combination with growth factors (PDGF, VEGF, FGF, PRF, L- PRF, and mesenchymal stem cells) [23–25] or bone morphogenetic proteins (BMPs) for critical defects [16, 17, 20].

The use of heterogeneous inorganic bone such as BIO, BFL, and LBP, or grafts from bone bank (allogenic bone) in large defects, requires the association with other biomaterials to categorize the complex formed by the osteoconductive substitute, signaling substance (growth factors), and an osteoinductive substance (BMP), referred to as the triad of Marx [20, 21].

Even with lower NB values of bone substitutes from bovine hydroxyapatite (BIO, BFL, and LBP), the histological, histometric, and immunohistochemical parameters were very motivating in the LBP group, with the reversal of NB values at 40 days (LBP > BIO, *p* < 0.001). Until the last period, defects filled with this bone substitute also had particles filling the bone defect and active osteogenesis, supported by increased immunostaining of Runx2. Given that this group compared with the others showed a lower staining for OC, an important protein that denotes maturation of bone healing, there was still stimulation for bone tissue formation, probably maintaining the sequence of bone maturation. Therefore, further studies should be conducted in order to assess the osteoconductive behavior of LBP particles in critical defects over longer periods, 60 and 90 days, enabling greater indication of this alternative biomaterial to BIO.

A better result on bone formation was obtained with the CLN. Regardless of the analysis employed, this bone substitute was superior to the others evaluated in this study. In the first period, there was intense osteoblastic activity, observed through immunohistochemical analysis, which resulted in greater bone formation on the last assessed period, with almost total closure of critical bone defects. In the speculation about clinical applicability, maturation of bone tissue, an important factor for primary stability in the installation of dental implants [26, 27] demonstrated through immunohistochemistry, increased osteocalcin expression for the CLN group at 40 days compared to 14 days. This can be explained by the fact that 14 days is a recent period and this group is not yet chronologically present in the phase of calcification or maturation of bone tissue, since the bone neoformation process is still active. Consequently, at 40 days, the most advanced period, the maturation process becomes more evident.

Luvizuto et al. [28] investigated the osteoconductive properties of a *β*-tricalcium phosphate (BTCP) already established on the market (Cerasorb®), supplemented or not by BMP-2 in critical defects in rat calvaria. The authors found that, even without supplementation of BMP, BTCP presented very promising osteoconductive properties, including the defect closure with neoformed bone. In the review published by Damron [29] (2007), BTCP has been clinically proven as a suitable material for graft in the spinal region, periodontal defects, and orthopedic tumors.

As much as the field of tissue engineering constantly investigates the association of BTCP with other biomaterials, especially with growth factors for the reconstruction of bone defects [8], the results in this study showed that a bone substitute (CLN) with alloplastic origin was able to promote bone formation with superiority to heterogeneous biomaterials. In addition, with the purpose of exclusively evaluating the activity and potential of the bone substitute, biological membrane was not used in this study to avoid the influence of its osteopromotive effect or the periosteal removal provided by guided bone regeneration [30].

The limitations of this study were mainly related to the last investigated period of euthanasia, 40 days. In the face of biological responses, mainly from LBP, which showed remaining particles with osteoconductive activity, immunostaining for Runx2, and reduction of OC expression, an evaluation of later osteoconductive potential should be realized in future studies.

## 5. Conclusion

Based on the literature and on the results observed in this study, it was concluded that the CLN had the best osteoconductive behavior when used to fill critical defects created in rabbit calvaria. The LBP showed higher NB at 40 days when compared to the BIO and BFL groups and also showed osteogenic activity and preosteoblast in the last reporting period. At 40 days, the BFL showed the lowest osteoconductive property.

## Figures and Tables

**Table 1:** Statistical difference between source of variation time and group (two-way ANOVA test).

Variation factor	^*∗*^ *p*
Biomaterials	<0.001
Time	<0.001
Biomaterials × time	<0.001

*p* < 0.05 denotes statistical significance.

**Table 2:** Interactions among the biomaterials groups (Tukey test), independent of time.

Biomaterials (interactions)	Difference (%)	*p*	^*∗*^ *p* < 0.05 (significance)
CLN vs. BFL	23.56	<0.001	Yes
CLN vs. BIO	17.00	<0.001	Yes
CLN vs. LBP	16.79	<0.001	Yes
LBP vs. BFL	6.76	<0.001	Yes
LBP vs. BIO	0.20	0.934	No
BIO vs. BFL	6.56	<0.001	Yes

*p* < 0.05 denotes statistical significance.

**Table 3:** Interactions among the percentual values of new bone formation of biomaterials used at 14-day period (Tukey test).

Biomaterials (interactions)	Difference (%)	*p*	^*∗*^ *p* < 0.05 (significance)
CLN vs LBP	19.07	<0.001	Yes
CLN vs BFL	18.76	<0.001	Yes
CLN vs BIO	14.10	<0.001	Yes
BIO vs LBP	4.97	<0.001	Yes
BIO vs BFL	4.66	<0.001	Yes
BFL vs LBP	0.30	0.93	No

*p* < 0.05 denotes statistical significance.

**Table 4:** Interactions among the percentual values of new bone formation of biomaterials used at 40-day period (Tukey test).

Biomaterials (interactions)	Difference (%)	*p*	^*∗*^ *p* < 0.05 (significance)
CLN vs BFL	28.35	<0.001	Yes
CLN vs BIO	19.90	<0.001	Yes
CLN vs LBP	14.52	<0.001	Yes
LBP vs BFL	13.82	<0.001	Yes
LBP vs BIO	5.37	<0.001	Yes
BIO vs BFL	8.45	<0.001	Yes

*p* < 0.05 denotes statistical significance.

**Table 5:** Scores of immunohistochemical analysis established for experimental groups (Runx2, VEGF, and OC) at 14 and 40 days, showing light (1), moderate (2), or intense (3) immunostaining.

	Runx2		VEGF		OC	
	14 days	40 days	14 days	40 days	14 days	40 days
LBP	1	2	2	1	2	1
BFL	2	2	2	1	2	2
CLN	2	2	2	2	1	2
BIO	1	1	1	1	2	2

## Data Availability

The data used to support the findings of this study are included within the article.
